# Efficacy and safety of high-dose interleukin-2 treatment in patients with a history of brain metastases from renal cell carcinoma

**DOI:** 10.1186/2051-1426-3-S2-P129

**Published:** 2015-11-04

**Authors:** Ashwin Chandar, Ann W Silk, Joseph I Clark, Gregory A Daniels, David F McDermott, Michael Morse, Michael KK Wong, Mark Stein, Janice Mehnert, Shabbar Danish, Sandra Aung, Howard L Kaufman

**Affiliations:** 1 Rutgers-Robert Wood Johnson Medical School, New Brunswick, NH, USA; 2 Cancer Institute of New Jersey, Rutgers-Robert Wood Johnson Medical School, New Brunswick, NJ, USA; 3 Loyola University Medical Center, Division of Hematology Oncology, Maywood, IL, USA; 4 Moores Cancer Center, University of California San Diego, La Jolla, CA, USA; 5 The Cytokine Working Group; Division of Hematology/Oncology, Beth Israel Deaconess Medical Center, Boston, MA, USA; 6 Duke Cancer Center, Durham, NC, USA; 7 University of Southern California, Los Angeles, Los Angeles, CA, USA; 8 Department of Neurosurgery, Rutgers-Robert Wood Johnson Medical School, New Brunswick, NJ, USA; 9 Prometheus Laboratories Inc., San Diego, CA, USA

## Background

The efficacy and safety of high dose IL-2 therapy in patients with brain metastases due to renal cell carcinoma is not well characterized.

## Methods

Data were prospectively collected in a registry of 371 patients with RCC receiving high-dose IL-2, including 18 patients with a history of brain metastases who had undergone treatment for the brain metastases with radiation therapy or surgery. The median interval between the diagnosis of brain metastases and the start of IL-2 therapy was 7.0 months (range 1-116 months). Median overall survival in patients with brain metastases was 15.3 (95% CI 8.0 to not evaluable) months, as compared to 48.0 (95% CI 39.2 to 61.5) months in RCC patients without any history of brain metastases.

## Results

The response to IL-2 in the extra-cranial disease was assessed. In patients with brain metastases, 0/18 (0%) had a complete response, 1/18 (5.6%) had a partial response, and 4/18 (22.2%) had stable disease, whereas in patients without brain metastases, 15/353 (4.5%) achieved a complete response, 45/353 (13.4%) achieved a partial response, and 117/353 (34.8%) had stable disease. During the first course of IL-2, neurologic adverse events were reported in 6 subjects (2 events of confusion, 2 events of mental fatigue, 1 event of rigors, and 1 event of anxiety), but no seizures or intracranial hemorrhages were reported.

## Conclusions

While the efficacy of IL-2 in this population is limited, the neurologic adverse event rate is acceptably low; in carefully selected patients with brain metastases, IL-2 may be considered as a therapeutic option but response rates may be lower than in patients without CNS disease based on this limited cohort analysis.

